# Effectiveness of Three Types of Moisturizers on Senile Dry Skin: A Randomized Controlled Pilot Trial

**DOI:** 10.1155/2023/1809109

**Published:** 2023-07-20

**Authors:** Miku Aoki, Natsuki Hata, Junko Yotsuya

**Affiliations:** ^1^Division of Nursing, Faculty of Medical Sciences, University of Fukui, Fukui, Japan; ^2^Department of Nursing, Graduate School of Medical Sciences, University of Fukui, Fukui, Japan

## Abstract

**Background:**

For dry skin, the application of a hypoallergenic moisturizer twice daily is recommended in elderly individuals. However, it is not known which is the most effective and appropriate moisturizer among the commercially available moisturizers.

**Aims:**

In this study, we aimed to investigate the efficacy of the three widely used moisturizers for the treatment of senile dry skin. *Patients/Methods*. This interventional study involved elderly individuals aged >65 years who were living in a nursing home. The participants were randomly divided into the interventional (moisturizers A, B, and C) and conventional care groups. Moisturizers A, B, and C were applied on the skin of each member of the three intervention groups twice daily for 8 weeks. The water content of the stratum corneum and transepidermal water loss (TEWL) were evaluated before and after the intervention. Changes in these parameters among the groups were compared using two-way analysis of variance and a posthoc test.

**Results:**

Moisturizers A, B, and C and conventional care groups comprised six, seven, five, and four participants, respectively. The water content of the stratum corneum was significantly higher in the moisturizer A (*p* = 0.01) and B (*p* = 0.047) groups than in the conventional care group. There was no significant difference in TEWL among the groups.

**Conclusions:**

In terms of the appearance of the skin, white powder and small scales were both reduced in group A. Taken together with the water content, this was considered a clinically significant change.

## 1. Introduction

The skin of elderly people is affected by aging, resulting in a decline in the skin barrier function and changes in the skin structure. In the stratum corneum, the skin barrier function is impaired due to a decrease in the levels of differentiation-related proteins and the extracellular matrix, and the skin is prone to dryness. The prevalence of dry skin in elderly people is 45.3–55.6% [1–3]. Very dry skin causes itchiness, leading to scratching and cracks in the skin. Chronic itchiness may have an impact on the quality of life, and scratching can cause wounds or infection. Dry skin-related health problems cannot be overlooked. Moisturizing care is required to improve dry skin.

It is accepted that moisturizing care can help improve dry skin. Application of moisturizers [4–8] and standardized skin care regimens [9] have been shown to be effective in improving the symptoms of dry skin. According to a systematic review by Lichterfeld-Kottner et al., lipophilic leave-on products containing humectants decreased skin dryness and reduced the incidence of pruritus [10].

However, no studies have reported how to select a suitable moisturizer for elderly individuals from among the many moisturizers available commercially. In addition, care providers often notice no difference in the skin after the application of these moisturizers. Moreover, the type of moisturizing care, including the selection of an appropriate product and the frequency of application, which is the best for improving dry skin in the elderly, remains unclear. Therefore, the purpose of this study was to examine the efficacy of three of the most widely used moisturizers for treatment of dry skin in Japan.

## 2. Materials and Methods

### 2.1. Study Design and Setting

This interventional study was conducted at a nursing home with 100 beds in rural Japan from October 2019 to February 2020.

### 2.2. Participants

In this study, 39 healthy residents of a nursing home were recruited and screened for eligibility. The inclusion criteria were as follows: age >65 years, care staff or subject being aware of dryness of the lower leg, which is the target area, and provision of consent to participate in the study. The exclusion criteria were as follows: receiving treatment or skin care for skin diseases, skin injuries other than dryness of the skin in the target lower leg area, hypersensitivity to drugs and cosmetics, and a critical general condition.

### 2.3. Intervention

Three commercially available skin moisturizers were evaluated in this study. The contents of the moisturizers are shown in Table 1. Moisturizer A (Silty®) contained sericin and ceramide NP; moisturizer B (Batel^TM^ Moisturizing Lotion) contained squalene, cetyl-PG hydroxyethyl palmitamide (pseudoceramide), and arginine; and moisturizer C (SECURA Moisturizing Lotion) contained propylene glycol, vaseline, stearic acid, and cetearyl alcohol. Each product was applied to the lower limbs of the participants, twice a day for 8 weeks. All the products were applied on the participant's skin by the staff of the nursing home. To maintain the quality of moisturizing care, staff were instructed on how to apply the moisturizers. A maximum of two staff members applied a moisturizer to each participant. Participants in the conventional care group were asked to continue with their routine skin care. When staff felt skin dryness, they applied moisturizers after bathing. Moisturizer application was less than twice a week.

### 2.4. Outcome

The noninvasive biophysical skin assessments included the assessment of skin hydration and TEWL and were performed in each participant at baseline and after 8 weeks of therapy. Skin hydration was the main outcome, which was assessed using Mobile Moisture HP-10-N (Courage + Khazaka GmbH, Cologne, Germany). TEWL was assessed using VapoMeter SWL-5001 (Delfin Technologies, Kuopio, Finland). These parameters were measured in the skin of the lower extremities where the moisturizer was applied and in the skin of the upper extremity where the moisturizer was not applied. Considering the effect of bathing on the measured values, the measurements were taken on nonbathing days. The temperature and humidity could not be completely controlled because the measurements were taken at the facility. However, the participants were in the same room for 30 minutes before the measurement. In addition, the air conditioning in the facility was kept constant.

The appearance of the skin was evaluated according to the presence of (1) white powder and (2) small scales on the surface of the skin.

Information on participant characteristics such as age, sex, underlying disease, and the degree of nursing care required was recorded. The degree of nursing care was classified on a scale of 1 (requiring partial care in some areas of daily life) to 5 (requiring care in all areas of daily life) based on the Japanese nursing care insurance certification.

### 2.5. Sample Size

Sample size was calculated using G^*∗*^ Power 3.1.9.4. With random assignment to groups, independent samples of equal size, an alpha value 0.05, and power (1-beta) of 0.80, a sample of n=17 for each group would be necessary. It is possible to detect an effect size of 0.25 in repeated measures analysis of variance.

### 2.6. Randomization

After obtaining informed consent, the participants were randomly divided into four groups: moisturizers A, B, and C and conventional care groups. Considering the possibility of bias due to age, gender, care unit, and other factors, the stratified substitution block method was adopted to allocate the participants.

### 2.7. Statistical Method

Changes in the water content of the stratum corneum and TEWL were calculated by subtracting the preintervention value from the postintervention value. Changes were compared using two-way analysis of variance and a posthoc test (Dunnet method). The *α* value was 0.05. Statistical analysis was performed using SPSS ver. 26 (IBM Statistics).

### 2.8. Ethical Considerations

The study was conducted in accordance with the Declaration of Helsinki 1975 and was approved by the University of Fukui Medical School Research Ethics Review Committee (number 20190023). The purpose and objectives of the study, methods, freedom to discontinue participation, no disadvantage in medical care due to withdrawal or discontinuation, restrictions on the use of information for purposes other than those of the study, and guarantees of confidentiality and anonymity were explained verbally and in writing to the participants and their families, and written consent was obtained. We confirmed with the facility nurse that the physical condition of the patients was such that they could participate in the study, and the study was conducted in accordance with the inclusion criteria. During the study period, we carefully observed for any adverse events, and if any symptoms appeared, we provided appropriate treatment immediately.

## 3. Results

### 3.1. Participant Flow

**Figure 1:**
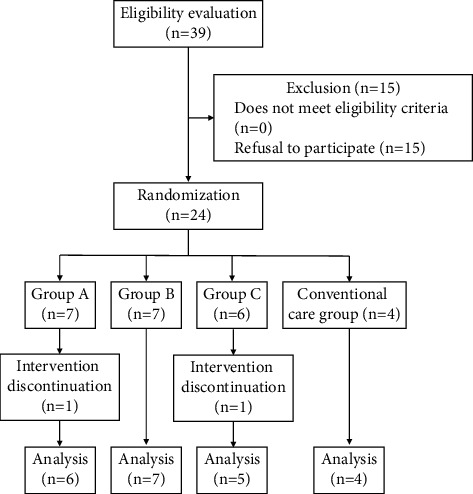
Participant flowchart of the study.

Figure 1 shows the participant flow. A total of 39 residents were initially screened for eligibility, of whom 15 declined to provide consent to participate; therefore, only 24 participants were assigned to the four groups. A rash was seen in one participant in the moisturizer A group. This rash was due to a pre-existing disease, and there was insufficient information on the screening of this disease. One participant in Group C was hospitalized during the intervention period. Therefore, these two participants were excluded from the analysis.

### 3.2. Participant Characteristics

Table 2 shows the baseline characteristics of the participants. The mean age ± SD was 84.8 ± 8.2, 85.6 ± 7.5, and 93.2 ± 3.0 years in the moisturizer A, B, and C groups, respectively, and 91.3 ± 3.3 years in the conventional care group. There was no difference in age, gender, underlying disease, or level of care required among the groups.

### 3.3. Outcome

The baseline parameters of the skin are shown in Table 3. At baseline, the moisture content was lower in the lower limbs than in the upper limbs in all groups. However, after 8 weeks, the intervention group had higher the moisture content in the lower limbs than in the upper limbs. Only in the conventional group, moisture content was lower in the lower limbs than in the upper limbs. TEWL was less in the lower limbs than in the upper limbs at baseline and 8 weeks in all groups.

**Figure 2:**
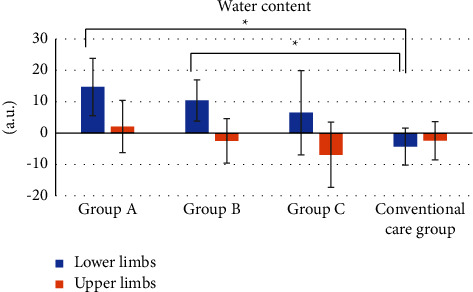
Changes in the water content of the stratum corneum. Data are expressed as mean ± SD. ^*∗*^*p* < 0.05.

Figure 2 shows the changes in moisture content in the stratum corneum. The moisture content was significantly higher in groups A (*p*=0.012) and B (*p*=0.047) than in the conventional care group. There was no significant difference among the groups in the upper limbs (to which moisturizer was not applied) (*p*=0.37).

**Figure 3:**
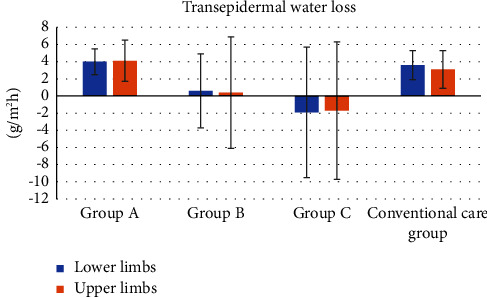
Changes in transepidermal water loss (TEWL). Data are expressed as mean ± SD.

Figure 3 shows the changes in TEWL. There was no significant difference among the groups in either the upper or lower limbs (*p*=0.15 and 0.34, respectively).

The changes in the skin appearance of the lower limbs are shown in Table 4. Groups A and B showed improvement in the white powder seen at baseline, while group C did not. On the other hand, small “dandruff”-like flakes were improved in all participants in groups A and C but not in those in group B and the conventional care group (60.0% and 100%, respectively).

## 4. Discussion

In this study, the application of moisturizers A and B significantly increased the moisture content of the stratum corneum. As there was no significant difference in the moisture content of the stratum corneum in the skin of the upper limbs (to which moisturizer was not applied) among the groups, the significant increase in the moisture content of the stratum corneum in the lower limbs can be attributed to the application of moisturizers. Although there was a significant difference in the water content only in groups A and B, the absolute values of water content in groups A, B, and C after 8 weeks indicate that the water content in the skin was sufficient. It was reported that water content increased from 31.1 to 34.4 to 32.7–37.3 when the structured skin care regimen was implemented on participants in an age similar to the subjects in our study (83.8 ± 8.3 years) [9]. The water content was about 10 (a.u.) greater in the participants in our study than in the previous study, suggesting that the moisturizers used in our study had a significant effect on increasing water content.

On the other hand, in TEWL, there was no significant difference among the groups or between the groups with and without moisturizer application. This suggests that the changes in TEWL were due to individual differences. There were group differences in the moisture content that may have been due to moisturizer application, but the effect was not sufficient to change TEWL. Previous studies have reported that TEWL is reduced by the application of moisturizers [4, 11]; the participants in those studies had a mean age of 73.5 ± 3.4 and 47 years, respectively. On the contrary, Hahnel et al. [9] reported an increase in TEWL after a 2-month application program of moisturizing cleansers and moisturizers. The participants in their study had a mean age of 83.8 ± 8.3 years, which is comparable to the age of participants in the present study (88.2 ± 6.7 years). A study reviewing differences in TEWL by age and the skin area reported that TEWL in individuals being 65 years and above was consistently lower compared to the group of 18–64-year-old individuals [12]. Chang et al. [13]. reported a change in TEWL from 3.14 to 2.81 after 15 days of skin care regimen on the lower legs of 63 (60–73)-year-old participants. Although the subjects were younger than in our study, they had lower TEWL. Among our study participants, there were differences in baseline TEWL, although not significant. This could be due to differences in conditions such as stratum corneum thickness. However, we did not obtain information on stratum corneum thickness in our study. Therefore, further investigation is needed to determine why TEWL did not show a significant difference, while water content showed a significant difference.

In terms of the appearance of the skin, white powder and small scales were both reduced in group A. Taken together with the water content, this was considered a clinically significant change. On the other hand, in groups B and C, the skin of not all of the participants showed improvement. From these results, it is considered that moisturizer A was the most effective among the three types used in this study. It is desirable to establish a standard for selecting moisturizers for the elderly people by further verification of skin conditions and moisturizing effects.

## 5. Limitations

This study has some limitations that should be considered. There were only a few participants (*n* = 4–7) in each group; the sample size was too small to mention statistically significant differences. In addition, the study included participants from one nursing home in a specific region; this limits the generalizability of our results.

## Figures and Tables

**Table 1:** Contents of the moisturizers used (INCI names).

Moisturizer	A (silty®)	B (Batel^TM^ moisturizing lotion)	C (SECURA moisturizing lotion)
Contents	Water, glycerin, dipropylene glycol, hydrogenated polyisobutene, methyl gluceth-10, sericin, ceramide NP, olea europaea fruit oil, polyquaternium-51, isohexadecane, ethylhexylglycerin, behenyl alcohol, glyceryl amide ethyl methacrylate/stearyl methacrylate copolymer, tetrasodium EDTA, xanthan gum, sodium acrylate/sodium acryloyldimethyl taurate copolymer, polysorbate 80, polyglyceryl-10 pentastearate, sodium stearoyl lactylate, stearyl glycyrrhetinate, acrylates/C10–30 alkyl acrylate crosspolymer, arginine, tetrasodium etidronate, butylene glycol, phenoxyethanol	Water, squalane, mineral oil, butylene glycol, glycerin, betaine, petrolatum, dimethicone, carbomer, butyrospermum parkii (shea butter), whey, olea europaea (olive) fruit oil, camellia japonica seed oil, sodium hyaluronate, cetyl-PG hydroxyethyl palmitamide, arginine, aloe barbadensis leaf extract, magnesium ascorbyl phosphate, steareth-6, sodium hydroxide, tetrasodium EDTA, methylparaben, ethylparaben	Purified water, propylene glycol, petrolatum, stearrate, cetearyl alcohol, ceteareth-20, glyceryl stearrate, aminomethyl propanol, acrylates/C10–30 alkyl acrylate copolymer, methylparaben, propylparaben

a.u., arbitrary units; TEWL, transepidermal water loss.

**Table 2:** Participant characteristics.

		Group A (*n* = 6)	Group B (*n* = 7)	Group C (*n* = 5)	Conventional care group (*n* = 4)	*p* value
Age, years	Mean ± SD	84.8 ± 8.2	85.6 ± 7.5	93.2 ± 3.0	91.3 ± 3.3	0.117

Sex, *n* (%)	Male	3 (50.0)	3 (42.9)	0 (0.0)	1(25.0)	0.293
	Female	3 (50.0)	4 (57.1)	5 (100.0)	3 (75.0)	

Underlying disease, *n* (%)	Dementia	0 (0.0)	4 (57.1)	3 (60.0)	2 (50.0)	0.121
	Heart disease	1 (16.7)	1 (14.3)	1 (20.0)	1 (25.0)	0.975
	Renal disease	0 (0.0)	1 (14.3)	1 (20.0)	1 (25.0)	0.668
	Diabetes	2 (33.3)	1 (14.3)	0 (0.0)	0 (0.0)	0.334
	Cerebral infarction	1 (16.7)	0 (0.0)	0 (0.0)	1 (25.0)	0.417

Degree of nursing care, *n* (%)	1	0 (0.0)	0 (0.0)	0 (0.0)	0 (0.0)	0.064
	2	1 (16.7)	0 (0.0)	2 (40.0)	0 (0.0)	
	3	2 (33.3)	4 (57.1)	2 (40.0)	0 (0.0)	
	4	3 (50.0)	3 (42.9)	1 (20.0)	2 (50.0)	
	5	0 (0.0)	0 (0.0)	0 (0.0)	2 (50.0)	

**Table 3:** Noninvasive parameters.

Water content (a.u.), mean ± SD	Baseline	Afer 8 weeks	Difference
*Lower limbs*			
Group A	33.6 ± 9.6	48.3 ± 8.3	14.7 ± 9.1
Group B	36.3 ± 5.6	46.6 ± 6.9	10.4 ± 6.6
Group C	38.8 ± 12.3	45.3 ± 3.2	6.5 ± 13.4
Conventional care group	35.8 ± 4.9	31.4 ± 8.5	−4.3 ± 5.9
*Upper limbs*			
Group A	40.8 ± 5.3	42.9 ± 7.6	2.1 ± 8.3
Group B	43.4 ± 7.3	40.9 ± 5.8	−2.5 ± 7.1
Group C	45.3 ± 8.1	38.5 ± 6.4	−6.9 ± 10.4
Conventional care group	42.1 ± 8.6	39.8 ± 8.3	−2.4 ± 6.1

*TEWL (g/m* ^*2*^ */h), mean ± SD*			

*Lower limbs*			
Group A	4.2 ± 1.2	8.2 ± 1.7	4.0 ± 1.5
Group B	8.3 ± 4.7	8.9 ± 1.8	0.6 ± 4.3
Group C	10.9 ± 7.2	9.0 ± 1.4	−1.9 ± 7.6
Conventional care group	5.2 ± 0.3	8.7 ± 1.9	3.6 ± 1.7
*Upper limbs*			
Group A	5.5 ± 2.0	9.6 ± 1.3	4.1 ± 2.4
Group B	10.0 ± 5.8	10.4 ± 2.8	0.4 ± 6.5
Group C	12.7 ± 7.3	11.0 ± 2.0	−1.7 ± 8.0
Conventional care group	8.5 ± 4.0	11.5 ± 1.9	3.1 ± 2.2

**Table 4:** Changes in skin appearance.

	Baseline	After 8 weeks	
	Yes	No	Yes
*White powder on the skin surface, n (%)*			
Group A	1 (16.7)	1 (100)	0
Group B	1 (14.3)	1 (100)	0
Group C	2 (40.0)	0	2 (100)
Conventional care group	0	—	—

*Small scales on the surface of the skin, n (%)*			
Group A	3 (50.0)	3 (100)	0
Group B	5 (71.4)	2 (40.0)	3 (60.0)
Group C	1 (20.0)	1 (100)	0
Conventional care group	2 (50.0)	0	2 (100)

## Data Availability

The data used to support the findings of this study are available from the corresponding author upon request.
